# 932. National Survey of Burn Advanced Practice Providers Roles and Responsibilities

**DOI:** 10.1093/jbcr/irag033.499

**Published:** 2026-04-06

**Authors:** Peter D Nguyen, Syed F Saquib, Michael de Virgilio, Areg Grigorian, Nicholas Truong, Patrick Murphy, Bellal Joseph, Marie Crandall, Rachael A Callcut, Mayur Patel, Andrew Bernard, Jeffry Nahmias

**Affiliations:** University of California Irvine, Orange, CA; University of California Irvine, Orange, CA; University of California Irvine, Orange, CA; University of California Irvine, Orange, CA; University of California Irvine, Orange, CA; Medical College of Wisconsin, Milwaukee, WI; University of Arizona Tucson, Tucson, AZ; MetroHealth, Cleaveland, OH; University of California Davis, Sacramento, CA; Vanderbilt University Medical Center, Nashville, TN; University of Kentucky, Lexington, KY; University of California Irvine, Orange, CA

## Abstract

**Introduction:**

Advanced practice providers (APPs) are essential contributors to burn care. Implementation and utilization of APPs has varied across burn centers and regions. Therefore, their clinical roles and responsibilities are not well defined. This study aimed to characterize APP utilization across burn surgery nationwide.

**Methods:**

A survey was distributed in December 2024 to the Acute Care Surgery (ACS) division chiefs through the Society of ACS Chiefs. This survey collected data on all ACS services (i.e., trauma, burns, emergency general surgery, and surgical critical care [SCC]) including burn surgery APP staffing, service coverage, procedural responsibilities, patient-to-APP ratios, onboarding practices, and educational support. Descriptive statistics were used to analyze the responses.

**Results:**

Of 74 division chiefs surveyed, 57 (77%) responded, with only 24 (32% of all surveys sent and 42% of all respondents) having a dedicated burn surgery service. Most respondents with a burn surgery service were university-based (62%) and Level-I (79%) trauma centers. Among the surveyed burn centers, 10 centers (42%) admitted >400 burn patients annually. Of those surveyed, 100% of burn centers had APPs, general surgery residents and SCC and/or ACS fellows. The mean number of APPs per burn surgery service was 1.8 (range 0-12). All centers reported some daytime APP coverage (mean APPs 1.3), but only 14 centers (58%) had nighttime APP coverage (mean APPs 0.3, range 0-3). Weekend APP coverage occurred in 88% of centers and 83% had holiday APP coverage. Less than half (46%) of surveyed centers reported burn surgery APPs to function independently. Burn surgery APP procedural involvement included assisting in the operating room (79%), arterial lines (58%), central lines (54%), and rarely intubations (8%). The median APP onboarding period was 12 weeks, and only 2 programs (8%) offered a dedicated burn surgery APP residency/fellowship.

**Conclusions:**

APPs are integrated into many university-based burn surgery services, with all centers surveyed having daytime APP burn coverage but only slightly more than half had nighttime APP coverage.

**Applicability of Research to Practice:**

Standardizing APP roles, onboarding, and procedural training may improve consistency and care for critically ill burn patients.

**Funding for the study:**

N/A.

